# The prostaglandin receptor EP2 determines prognosis in EP3-negative and galectin-3-high cervical cancer cases

**DOI:** 10.1038/s41598-020-58095-3

**Published:** 2020-01-24

**Authors:** Sebastian Dietlmeier, Yao Ye, Christina Kuhn, Aurelia Vattai, Theresa Vilsmaier, Lennard Schröder, Bernd P. Kost, Julia Gallwas, Udo Jeschke, Sven Mahner, Helene Hildegard Heidegger

**Affiliations:** 1 Department of Obstetrics and Gynecology, LMU Munich, University Hospital, Campus Innenstadt, Munich, Germany; 2 Department of Obstetrics and Gynecology, LMU Munich, University Hospital, Campus Großhadern, Munich, Germany

**Keywords:** Immunohistochemistry, Cervical cancer

## Abstract

Recently our study identified EP3 receptor and galectin-3 as prognosticators of cervical cancer. The aim of the present study was the analysis of EP2 as a novel marker and its association to EP3, galectin-3, clinical pathological parameters and the overall survival rate of cervical cancer patients. Cervical cancer tissues (n = 250), as also used in our previous study, were stained with anti-EP2 antibodies employing a standardized immunohistochemistry protocol. Staining results were analyzed by the IRS scores and evaluated for its association with clinical-pathological parameters. H-test of EP2 percent-score showed significantly different expression in FIGO I-IV stages and tumor stages. Kaplan-Meier survival analyses indicated that EP3-negative/EP2-high staining patients (EP2 IRS score ≥2) had a significantly higher survival rate than the EP3-negative/EP2-low staining cases (p = 0.049). In the subgroup of high galectin-3 expressing patients, the group with high EP2 levels (IRS ≥2) had significantly better survival rates compared to EP2-low expressing group (IRS <2, p = 0.044). We demonstrated that the EP2 receptor is a prognostic factor for the overall survival in the subgroup of negative EP3 and high galectin-3 expressed cervical cancer patients. EP2 in combination with EP3 or galectin-3 might act as prognostic indicators of cervical cancer. EP2, EP3, and galectin-3 could be targeted for clinical diagnosis or endocrine treatment in cervical cancer patients, which demands future investigations.

## Introduction

Cervical cancer is the fourth most common female cancer type worldwide with nearly half a million new cases annually. 83% of all cases occur in developing countries, whereas in developed countries only 3.6% of new cancer cases are cervical cancer^1^. One of the main risk factors for cervical cancer is a persistent infection with specific Human Papillomavirus (HPV), the high-risk papillomavirus (HR-HPV)^2^. HPV is manifested in nearly 99.7% of all cervical cancer patients^3^. Belonging to the papillomavirus family, HPV is a non-enveloped, small, double-stranded DNA virus^3–5^. More than 200 HPV genotypes have been characterized worldwide^6^. The sub-classification in high-risk and low-risk is important for the HPV infections of the genital tract^5^. Low-risk subtypes, such as HPV-6, HPV-11, HPV-26, HPV-40, HPV-42, are the cause of genital warts and non-malignant lesions^2,5^, whereas high-risk HPV types like HPV-16 and HPV-18 were identified in cervical and other anogenital cancers^2,5^.

Tumor cell differentiation, apoptosis, and oncogenesis are associated with prostanoids, including prostaglandin E_2_ (PGE_2_), prostaglandin D_2_ (PGD_2_), prostaglandin I_2_ (PGI_2_), prostaglandin F_2_ (PGF_2_) and thromboxane A_2_^7^. Prostaglandins are important for tumor progression and tumor-associated angiogenesis as Amano *et al*. described^8^. PGE_2_ signaling is well-known for apoptosis inhibition, angiogenesis, metastatic formation, and tumor progression. The membrane-bound EP receptors specific for PGE_2_ are G-protein coupled receptors and are classified into four subtypes: EP1, EP2, EP3, and EP4. Sales *et al*. showed the up-regulated expressions of PGE_2_, EP2 and EP4 in cervical cancer tissues compared to in normal cervical tissues, indicating that PGE_2_ may regulate neoplastic cell function in cervical carcinoma via EP2 and EP4 receptors^9,10^. Additionally, we previously reported that the EP3 receptor is an independent negative prognosticator for cervical cancer patients^11^. However, it remains unknown whether EP2 expression is correlated to the overall survival rate of cervical cancer patients and its correlation with other clinical-pathological parameters.

Galectins are members of a family of β-galactosidase binding animal lectins^12–14^, and play a vital role in cancer progression and metastasis^15,16^. Galectin-3 may play a crucial role in cell adhesion, cell growth and apoptosis in cancer development^17–19^. Altered galectin-3 expression is correlated to the stage of tumor progression in many types of carcinoma, such as colon, thyroid, breast and prostate cancer^20–23^. There are only a few reports concerning the role of galectin-3 in cervical cancer, and literature about the relation between galectin-3 and cervical cancer is limited. Li *et al*. showed that galectin-3 is a risk factor for the survival rate in cervical cancer patients^24^. Our study previously demonstrated that galectin-3 expression is correlated with a poor prognosis in the overall survival analysis of cervical cancer patients with no or low p16 expression^25^.

The aim of this study was to analyze EP2 expression in human squamous cell carcinoma (SCC) and adenocarcinoma (AC) of the cervix in relation to overall survival and to investigate whether EP2 is associated with EP3 and galectin-3 regarding the survival of cervical cancer patients.

## Material and Methods

### Clinical pathologic characteristics

Two hundred fifty cervical cancer specimens were obtained from the Department of Obstetrics and Gynecology at the Ludwig-Maximilians-University in Munich, Germany. These cervical cancer patients underwent surgeries between the years 1993 and 2002 and formalin-fixed paraffin-embedded (FFPE) samples used for immunohistochemistry tests were histopathological tumor samples. The patients’ median age was 47.0 years (range 20–83 years); the overall median survival was 100 months. The distribution of clinic-pathological variables can be seen in Table 1. Only patients with adenocarcinoma or squamous cell carcinoma of the cervix were used in our study; other histological subtypes were excluded because of the low number of patient cases and different tumor biology. We applied placenta tissues as the positive and negative controls for EP2 immunohistochemical staining, and they were also received from the Department of Obstetrics and Gynecology of the Ludwig-Maximilians-University in Munich (Supplementary Fig. 1).

**Table 1:** Clinical-pathological variables of the patients included in the study.

Item	Numbers/Total Numbers	Percentage (%)
**Age**		
<49	139/250	55.6
>49	111/250	44.4
**Number of positive lymphnodes**		
0	151/250	60.4
>1	97/250	38.8
not available	2/250	0.8
**pT, Tumor size**		
pT1	110/250	44.0
pT2/3/4	137/250	54.8
not available	2/250	0.8
**FIGO**		
I/II	112/250	44.8
III/IV	44/250	17.6
not available	94/250	37.6
**Tumor grade**		
I	21/250	8.4
II	143/250	57.2
III	78/250	31.2
not available	8/250	3.2
**Tumor subtype**		
squamous	202/250	80.8
adenocarcinoma	48/250	19.2
**Progression (over 235 months)**		
none	210/250	84.0
at least one	21/250	11.6
not available	11/250	4.4
**Survival (over 235 months)**		
right censured	190/250	76.0
died	49/250	19.6
not available	11/250	4.4

Cervical cancer tissues (n = 250), as also used in our previous study, were stained with anti-EP2 antibodies employing a standardized immunohistochemistry protocol. Staining results were analyzed by the IRS scores and evaluated for its association with clinical-pathological parameters.

This study was approved by the ethical committee of the Medical Faculty, Ludwig-Maximilian-University of Munich (approval number: 259-16). All methods were performed in accordance with the standards set in the declaration of Helsinki 1975. Staging and grading were assessed by two gynecological pathologists according to the criteria of FIGO and WHO. Follow-up data were received from the Munich Cancer Registry (Munich Tumour Center, Munich, Germany) for statistical analyses of 248 cervical cancer patients. Patients’ samples and clinical information were anonymized and encoded for statistical workup. All clinical information was blinded from the authors during experimental analysis.

### Immunohistochemistry (IHC)

The FFPE samples were stored at room temperature, cut (3 µm) from all specimens and attached to positively charged microscope slides. After being in xylol for 20 minutes to remove paraffin, tissues were washed in pure ethanol and endogenous peroxidase and then blocked with 3% methanol/H_2_O_2_ solution for 20 minutes. The tumor slides were rehydrated in a descending alcohol series. To unmask the antigen after formalin-fixation-associated protein-agglomeration, all slides were heated up to 100 °C in a pressure cooker containing trisodium citrate buffer solution (Merck 244 and Merck 6448) with pH = 6.0 and cooked for 5 minutes at 100 °C. After rinsing in distilled water and PBS, slides were stained by Reagent 1 of polymer detection kit (ZytoChem Plus HRP Polymer System, Mouse Rabbit, Zytomed) for 5 minutes to avoid unspecific primary antibodies binding. Then samples were incubated with the EP2 primary antibody (anti-PTGER2 antibody polyclonal rabbit IgG; Abcam ab 189028, Lot No. GR 246965-4) for 16 hours at 4 °C. After washing in PBS, slides were stained with a post-block-reagent and secondary antibodies conjugated with horseradish peroxidase (HRP). The substrate-staining with the DAB (chromogen substrate kit, Dako) were counterstained for 2.5 minutes and afterward with hematoxylin for 2 minutes. The samples were then dehydrated in a rising alcohol series and covered with the mounting medium Eukitt.

### IHC controls

FFPE sections of human placental tissue were used as either positive or negative control tissue of EP2 staining and were picked after searching the Human Protein Atlas (Supplementary Fig. 1)^26^. Negative controls were performed by replacement of the primary antibodies by species specific isotype control antibodies (Dako) as described previously^27^. In addition, the IgG negative control was performed with unspecific isotype control IgG antibodies in Supplementary Fig. 1.

### Evaluation of IHC staining

Two independent blinded observers rated the expression intensity and distribution pattern of the immunohistochemical staining in tumor samples by using the immunoreactive score (IRS) under a Leitz (Wetzlar, Germany) microscope. The concordance before the re-evaluation was 98.8% (n = 247/250) and 0.12% (n = 3/250) diverging cases were revised to have the same result. This semi-quantitative IRS score was calculated by multiplication of the staining intensity (0 = not stained; 1 = low intensity; 2 = moderate intensity; 3 = high intensity) and the percentage of stained cells (0 = 0%; 1 = 1–10%; 2 = 11–50%; 3 = 51–80%; 4 ≥ 80%). The EP2 percentage score (EP%) used in the nonparametric correlation analysis is defined as staining intensity (0 = not stained; 1 = low intensity; 2 = moderate intensity; 3 = high intensity) multiplied with the percentage of stained cells from 0–100% in 0, 5, 10, 20, 30, 40, 50, 60, 70, 80, 90, 100% intervals^28^. The IRS score focuses on the staining intensity, while the percentage score focuses on the smaller percentage intervals of stained tumor cells to differentiate in similar staining intensity. The EP2 expression level was analyzed through semi-quantitative scoring (n = 248, two patients were not included in this analysis because of the missing follow-up data).

### Statistical analysis

We used IBM statistical SPSS (version 24) for statistical analysis. ROC analysis, Chi-square test, and Spearman’s correlation analysis were performed. Survival times were compared by Kaplan–Meier graphics and differences in overall patient survival were tested for significance by using Chi-square statistics of the log-rank (Mantel-Cox) test. Data were assumed to be statistically different in the case of p < 0.05.

### EP2 receptor isoforms

The anti-prostaglandin E_2_ receptor EP2 antibody-N-terminal (ab189028, Abcam Lot: GR246965-4) binding site is the 18 amino acid peptide from N-terminal extracellular domain NP_000947.2 with the amino acid sequence (ATQTSCSTQS DASKQADL) positioned from 340 to 358. Abcam BLAST analysis of the peptide immunogen showed no homology with other Human proteins^29^. EP2 mutated isoforms 1–87 were analyzed: all six cervical cancer mutations have the N-terminal extracellular domain site of the anti-EP2 antibody we used. Three mutated amino acid chain isoforms of EP2 cannot be stained with this anti EP2 antibody because of the missing N terminal binding site (1: Genome Position chr14:52794146-52794146 in skin cancer (AA) position 351 Reference D to Variable N, 2: Genome Position chr14:52794167-52794167 in lung cancer AA 358 Reference L to Variable F, 3. Genome Position chr14:52794168-52794168 in liver cancer AA 358 Reference L to Variable P)^30^. Therefore, we are convinced that the functional (non-N-terminal mutated) EP2 receptor was stained in our cervical cancer tissues.

### Ethical approval

The tissue samples used for the analysis presented here were classified as leftover material. The samples had been collected for routine diagnostics at the time the patient was treated at our institution. When this retrospective study was initiated all diagnostic procedures had already been fully completed and the tissue underwent irreversible anonymization. Tissue samples were retrieved from the archive of Gynecology and Obstetrics, Ludwig-Maximilians-University, Munich, Germany. The local ethics committee of the Ludwig-Maximilians University approbated the ethical vote of this study (reference number: 259-16, 13rd of June 2016, Munich, Germany) and the Helsinki Declaration guidelines were respected.

## Results

### The percentage of EP2 expression is significantly decreased from FIGO I to FIGO II

**Figure 1:**
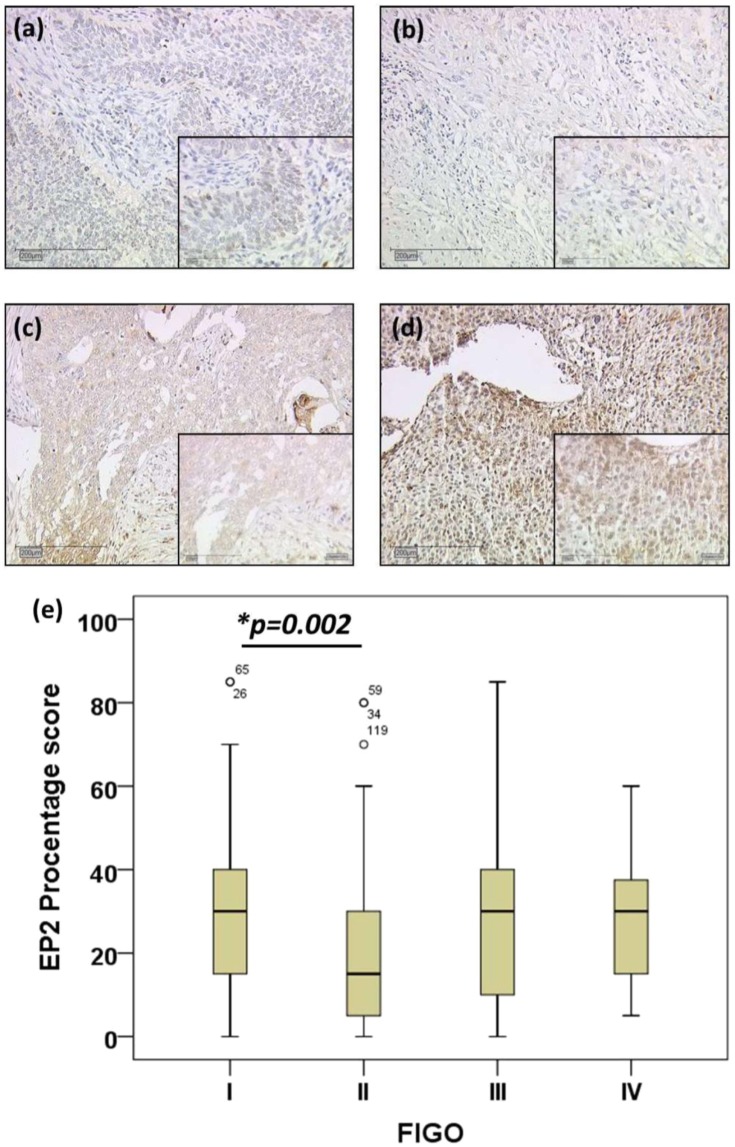
EP2 expression shown as the percentage of stained tumor cells compared to FIGO classification. This Figure shows immunohistochemistry (IHC) microphotographs of cervical cancer patients with a magnification of 10× and an insert of 25×. IHC patient with a tumor grade of FIGO 1 and EP2 staining of 30 percent stained tumor cells (**a**); IHC patient with a tumor grade of FIGO 2 and EP2 staining of 15 percent stained tumor cells (**b**); IHC patient with a tumor grade of FIGO 3 and EP2 staining of 40 percent stained tumor cells (**c**); IHC patient with a tumor grade of FIGO 4 and EP2 staining of 50 percent stained tumor cells (**d**); H-test Boxplot EP2 percentage of stained tumor cells compared to the FIGO grading 1–4 (p = 0.015) (**e**).

We applied the same cervical cancer tissues as were used in our previously published studies^11^. EP2 was stained in the cytoplasm of 94.8% cervical cancer tissues (235/248), consisting of 188 cases with squamous cell carcinoma and 47 cased with AC. Nonparametric correlation analysis showed neither EP2 IRS score nor EP2 percentage score was correlated to FIGO staging (p = 0.369, correlation coefficient = −0.057; p = 0.287, correlation coefficient = −0.068; respectively, Table 2). However, the H-test showed the significant EP2 percentage of stained tumor cells differed in four different groups of summarized FIGO gradings I-IV (p = 0.015, Fig. 1e). The median of EP2 staining percentage score was 30% in cervical cancer patients with FIGO I (n = 64), 15% in patients with FIGO II (n = 49), 30% in patients with FIGO III (n = 37) and 30% in patients with FIGO IV (n = 7, Fig. 1e). EP2 staining in patients with FIGO II was significantly decreased compared to cases with FIGO I (p = 0.002, Fig. 1e). The IHC cytoplasmic staining pattern of FIGO I SCC (Fig. 1a), FIGO II SCC (Fig. 1b), FIGO III adenocarcinoma (Fig. 1c) and FIGO IV SCC (Fig. 1d) were shown as examples of each FIGO grading group.

**Table 2:** Correlation analysis.

Variables	EP2 IRS score		EP2 percentage score	
	p Value	Correlation coefficient	p Value	Correlation coefficient
pT	0.81	−0.015	0.648	−0.029
pN	0.917	−0.007	0.601	−0.033
pM	0.212	−0.079	0.199	−0.082
Histotype	0.3	−0.066	0.328	−0.062
Grading	0.561	0.037	0.524	0.041
FIGO	0.369	−0.057	0.287	−0.068
EP3	<0.01*	0.232	<0.01*	0.278
galectin−3	0.06	0.121	0.016*	0.154
E6	0.843	−0.013	0.989	−0.001
p16	0.184	−0.087	0.062	−0.122
p53	0.348	0.06	0.408	0.053
MDM2	0.852	0.012	0.776	0.018
GPER	0.817	0.015	0.36	0.058

pT, tumor status; pN, lymph node status, pM, metastasis status, FIGO, the International Federation of Gynecology and Obstetrics; GPER, G protein-coupled estrogen receptor.

**Figure 2:**
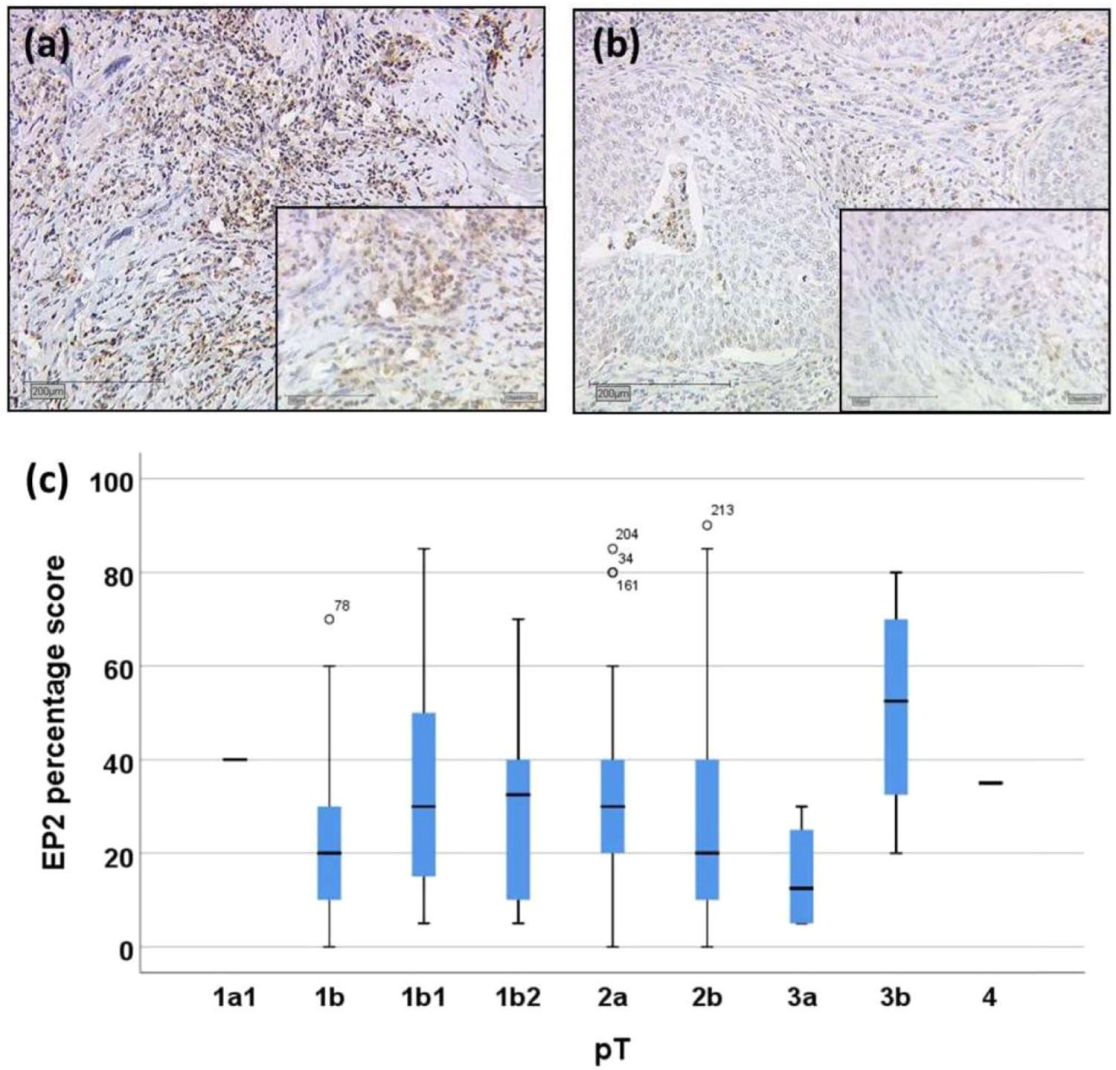
EP2 immunohistochemistry (IHC) microphotographs of cervical cancer patients related to tumor staging (pT) with a magnification of 10× and an insert of 25×. IHC with a tumor grade of pT 1b1 and EP2 staining of 30 percent stained tumor cells (**a**); IHC with a tumor grade of pT T2b and EP2 staining of 20 percent stained tumor cells (**b**); H-test Boxplot EP2 percentage of stained tumor cells compared to tumor grading pT with subtypes (p = 0.023) Median percentage of EP2 stained tumor cells are in pT 1a1 (n = 1) has 40%, pT 1b (n = 55) 20%, pT 1b1 (n = 44) 30%, pT 1b2 (n = 12) 35%, pT 2a (n = 28) 30%, pT 2b (n = 100) 20%, pT 3a (n = 4) 15%, pT 3b (n = 4) 50%, and pT 4 (n = 1) 35% (**c**).

Nonparametric correlation analysis showed pT staging was not correlated to neither EP2 IRS score (p = 0.81, correlation coefficient = −0.015, Table 2) nor EP2 percentage score (p = 0.648, correlation coefficient = −0.029, Table 2). However, the H-test showed significant changes in the percentage of EP2-expression in eleven different groups of TNM primary tumor stages (p = 0.023, Fig. 2c). The median of EP2 percentage score was increased with pT1 stage: 20% in pT 1b (n = 55), 30% in pT 1b1 (n = 44) and 35% in pT 1b2 (n = 12). The median of EP2 percentage score was decreased with higher pT stages until pT 3a: 30% in pT 2a (n = 28 cases), 20% in pT 2b (n = 100 cases) and 15% in pT 3a (n = 4 cases). The median of EP2 percentage staining was 50% in four cervical cancer patients with pT 3b and 35% in one patient with pT 4, respectively. Figure 2a showed an example of 30% cytoplasmic EP2 percentage expression with pT 1b1, and Fig. 2b showed 20% cytoplasmic EP2 percentage expression with pT 2b.

In summary, EP2 showed significant changes connected to tumor staging (pT) and FIGO. EP2 staining was increased with pT1 stage and decreased from pT 2a until pT 3a (Fig. 2c). In addition, EP2 staining in patients with FIGO II was significantly decreased compared to cases with FIGO I (Fig. 1e). These suggest that EP2 might be a novel bio-marker for cervical cancer.

### Nonparametric correlation

Nonparametric correlation analysis indicated a significant correlation of EP2 percentage score with galectin-3 IRS score (p = 0.016; correlation coefficient = 0.154, Table 2). EP3 expression was correlated to both EP2 IRS score (p < 0.01; correlation coefficient = 0.232, Table 2) and EP2 percentage score (p < 0.01; correlation coefficient = 0.278, Table 2) in cervical cancer patients. However, EP2 IRS staining was not associated to tumor histology, pT, lymph node status (pN), metastasis status (pM), grade or FIGO stages in our database (Table 2). The staining of the proteins was performed on the same patients and tumor samples.

Immunohistochemical receptor staining intensities of E6, p16^25^, p53, cytoplasmic p53, mutant p53, mutant cytoplasmic p53^31^, MDM2^25^, cytoplasmic G protein-coupled estrogen receptor (GPER)^32^, nuclear receptor-interacting protein 1 (NRIP1)^33^ also were not linked to EP2 staining intensity.

### Overall survival rate analysis combining EP2 and EP3

The cut off value of EP2 staining was analyzed by ROC and defined at 28% of stained tumor cells. High expression of EP2 staining was defined by IRS ≥2 (n = 114 cases) and low EP2-expression at the value of IRS <2 (n = 133 cases). High EP2 expression (IRS ≥2) and low EP2 expression (IRS <2) did not differ in the overall survival rate of 250 cervical cancer patients (p = 0.361, data not shown).

**Figure 3:**
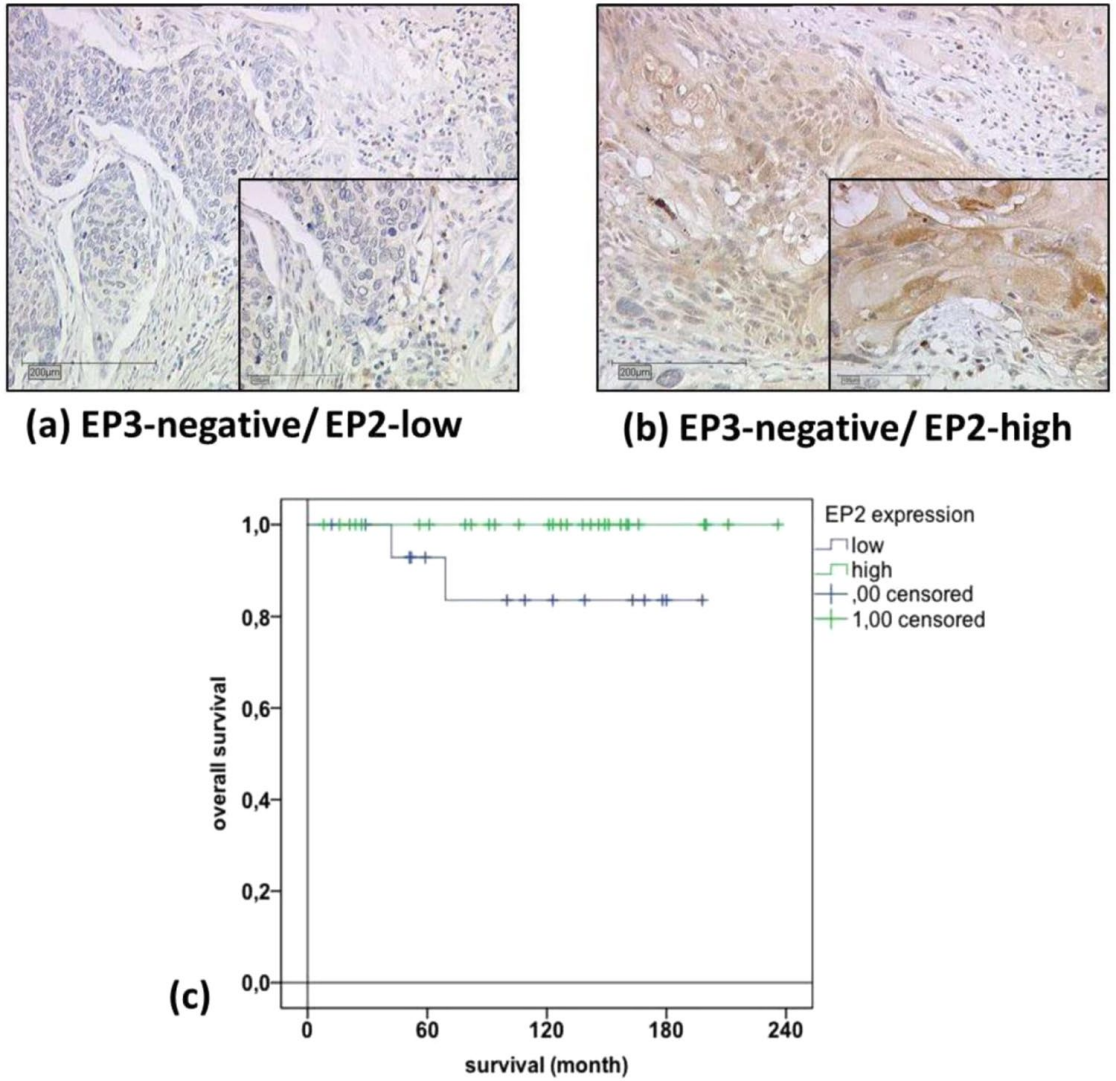
EP3 IRS 0 stained squamous cell carcinoma patient (**a**) and EP2 IRS 8 stained squamous cell carcinoma patient (**b**) is shown. Serial section of the same patient, with a magnification of 10× and an insert of 25×; Kaplan-Meier curve: survival function of EP3 IRS 0 stained patient group with EP2 high (green) IRS >2 and low (blue) IRS <2 expression (**c**).

For this combined analysis we used the whole number of patients enrolled in our study (n = 250), no pre-selection was done. Our previous study demonstrated that high EP3 expression was associated with a poor overall survival rate of the same patients^11^. In the subgroup of cervical cancer patients with no EP3 expression (IRS score = 0, n = 45/250, 18%), the survival rate differed in the high-EP2 (n = 29/45, 64%) (Fig. 3b) and low-EP2 expression group (n = 16/45, 36%) (Fig. 3a). The EP3 unstained patients with high EP2 expression had significantly higher survival rates than the low stained EP2 patients (p = 0.049; chi-square 3.888, Fig. 3c). All of the EP3-negative/EP2-high expression patients survived after 20 years’ observation time, whereas the EP3-negative/EP2-low expression group displayed reduced survival rates in the Log-rank (Mantel-Cox) test (Fig. 3c). The results suggested that EP2 is a positive prognostic factor for overall survival in EP3-negative cases.

We analyzed also the relationship between EP2 staining and the FIGO stage or the tumor stage in the EP3- unstained subgroup. There we could not find any significant results. In the correlation analysis in the high galectin-3 subgroup we found a significant correlation between EP2 IRS score and EP3 IRS score (p < 0.001).

### Overall survival rate analysis combining EP2 and galectin-3

**Figure 4:**
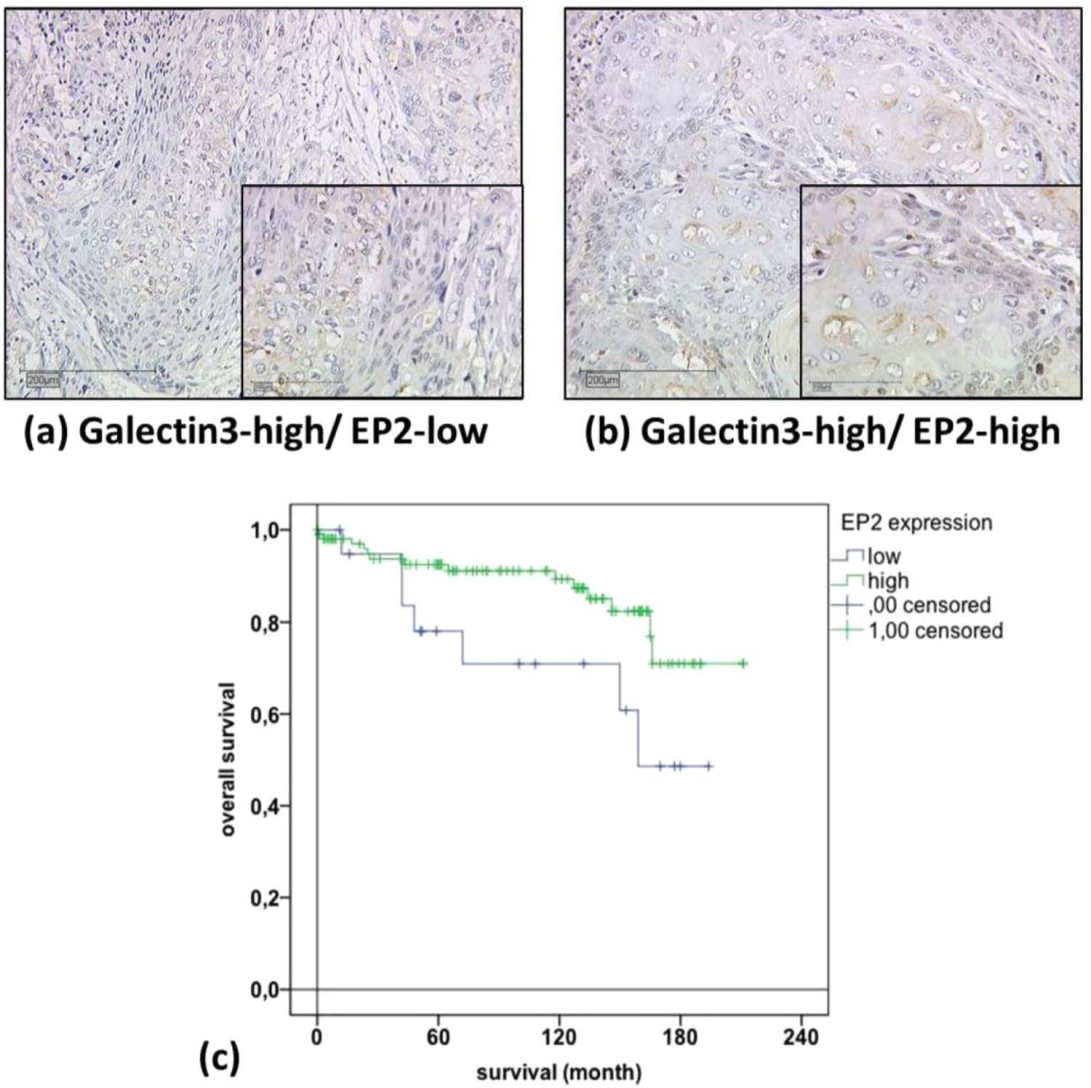
Galectin-3 (gal-3) staining (**a**) and EP2 staining (**b**) is shown. Serial section of the same patient are shown, with a magnification of 10× and an insert of 25×: gal-3 IRS >3 stained squamous cell carcinoma patient (**a**); EP2 IRS >2 stained squamous cell carcinoma patient (**b**); Kaplan-Meier curve: survival function of gal-3 IRS >3 stained patient group compared with EP2 high (green) IRS >2 and low (blue) IRS <2 expression (**c**).

In addition, another study by our research group proved that galectin-3 expression was correlated with poor prognosis in p16-negative cervical cancer patients. In cases with high galectin-3 expression (galectin-3 IRS score >3, n = 125/250, 50%), the EP2-high staining group (n = 105/125, 84%) (Fig. 4b) had significantly better survival rates than the EP2-low staining group (n = 20/125, 16%) (Fig. 4a) in the Log-rank (Mantel-Cox) test (p = 0.044; chi-square 4.075, Fig. 4c).

We analyzed also the relationship between EP2 staining and the FIGO stage or the tumor stage in the high galectin-3 subgroup. There we could not find any significant results. In the correlation analysis in the high galectin-3 subgroup we found a significant correlation between EP2 IRS score and EP3 IRS score (p  <0.001).

## Discussion

This study describes that high EP2 staining is a prognostic factor in low EP3 and high gal-3 stained cervical cancer patients. In this study, we observed that EP2 in combination with negative EP3 or high galectin-3 was a prognostic factor for survival in cervical cancer patients.

The majority of studies indicate that EP2 is beneficial for carcinoma development. EP2 overexpression is correlated with worse prognosis in esophageal squamous cancer^34,35^, and tumoral microsatellite instability in colorectal cancer^36^. Tumor growth is inhibited in EP2-knockout mice compared with wild-type mice^37–39^. Asting *et al*. showed that tumor growth, systemic inflammation and the expression of interleukin-6 are reduced in EP2-knockout tumor-bearing mice^40^. Tian *et al*. found that EP2 methylation is associated with a better prognosis of non-small cell lung cancer, and EP2 methylation is present with greater frequency in tumors with epidermal growth factor receptor (EGFR) mutation than in non-EGFR mutated tumors^41^. This indicates that EP2 methylation silences the expression of the gene thus demonstrating that an absence of EP2 protein is correlated with improved prognosis. Sales *et al*. demonstrated that PGE_2_ regulates the function of cervical carcinoma cells mainly via cyclic adenosine monophosphate (cAMP) linked EP2/EP4 signaling pathway^9,10^. Chang *et al*. found that the EP2 pathway (EP2-Gs-cAMP-protein kinase A) is required for cyclooxygenase 2-mediated mammary hyperplasia^42^. In the current study, we observed that high EP2 levels (IRS ≥2) had significantly better survival rates compared to EP2-low expressing group in the subgroup of EP3-negative or high galectin-3 expressing patients. The majority of studies state that EP2 is beneficial for tumor genesis, however, combining EP2 and EP3 or EP2 and galectin-3 showed the converse outcome in cervical cancer. This suggests that crosstalk might exist in them, therefore, EP2/EP3/galectin-3 signaling pathway would be analyzed in further *in vivo* investigations with cervical cancer cell lines (HeLa, CaSki, Siha and C-33A).

We found that association of the EP2 receptor with either high galectin-3 or negative EP3 expression cervical cancer patients resulted in better survival in both subgroups, respectively. Different isoforms of EP2 receptor might be another factor resulting in the outcomes of the current study. Many details of the EP2 receptor and its isoforms are yet to be discovered and the available data shows some discrepancies, especially concerning its effects. The isoforms of the EP2 receptor may have different effects and physiological roles based on the tissue, in which they are expressed. Further investigation is needed to understand the downstream signaling pathway and its effects on tumor growth and invasiveness and their links to survival.

It is known that EP2 coupling with G protein alpha stimulator to increase cAMP production while EP3 coupling with G protein inhibitor to decrease cAMP production. The EP3 receptor contributes to malignant aggressiveness, carcinogenesis and poor prognosis in several cancer types like lung adenocarcinoma, endometrial carcinoma^43^ and breast carcinoma^44^. Another study observed that upregulation of EP3 expression in prostate cancer cells is associated with preventive and anticancer effects^45^. We observed that the prostaglandin EP3 receptor was an independent negative prognostic factor for cervical cancer^11^.

Previous studies found a correlation between galectin-3 and the development of cervical cancer. Li *et al*. showed a poor prognosis in cervical cancer patients with overexpression of galectin-3 protein^24^. In contrast, Lee *et al*. suggested that downregulation of galectin-3 in cervical cancer tissues is associated with the progression of cervical cancer^46^. In our previous studies, Stiasny *et al*. showed that galectin-3 was a negative independent prognosticator for the overall survival of patients with p16-negative cervical cancer^25^. Therefore, we were able to compare the results of the recent study with both previous studies on EP3 and galectin-3, respectively. Within this study we observed that of EP2 percentage score correlates to galectin-3 with immunohistochemical evaluation, possibly indicating a link between the regulation of EP2 and galectin-3 expression in cervical cancer tumor cells.

## Conclusion

In the present study, we could observe that the EP2 receptor in combination with high galectin-3 or negative EP3 was a significant prognostic factor for survival in cervical cancer patients. For the future, targeting the EP2 receptor as a means of diagnosis or therapy seems possible, but more research is needed to understand the exact relations of the prostaglandin receptor system and cervical cancer.

## Supplementary information

Supplementary Figure 1.
