# Supplementary material for: The prostaglandin receptor EP2 determines prognosis in EP3-negative and galectin-3-high cervical cancer cases
Source: Sci Rep. 2020 Jan 24;10:1154. doi: 10.1038/s41598-020-58095-3 (PMC6981231; doi:10.1038/s41598-020-58095-3)
Supplement: Supplementary file 1 — Supplementary Figure 1. [file 41598_2020_58095_MOESM1_ESM.docx]

**The prostaglandin receptor EP2 determines prognosis in EP3-negative and galectin-3-high cervical cancer cases**

Sebastian Dietlmeier ^1^, Yao Ye ^1^, Christina Kuhn ^1^, Aurelia Vattai ^1^, Theresa Vilsmaier ^1^, Lennard Schröder ^1^, Bernd P. Kost ^1^, Julia Gallwas ^1^, Udo Jeschke^1,2,*^, Sven Mahner^1,2^, Helene Hildegard Heidegger ^1^

^1^ Department of Obstetrics and Gynecology, LMU Munich, University Hospital, Munich, Germany, Campus Innenstadt

^2^ Department of Obstetrics and Gynecology, LMU Munich, University Hospital, Munich, Germany, Campus Großhadern


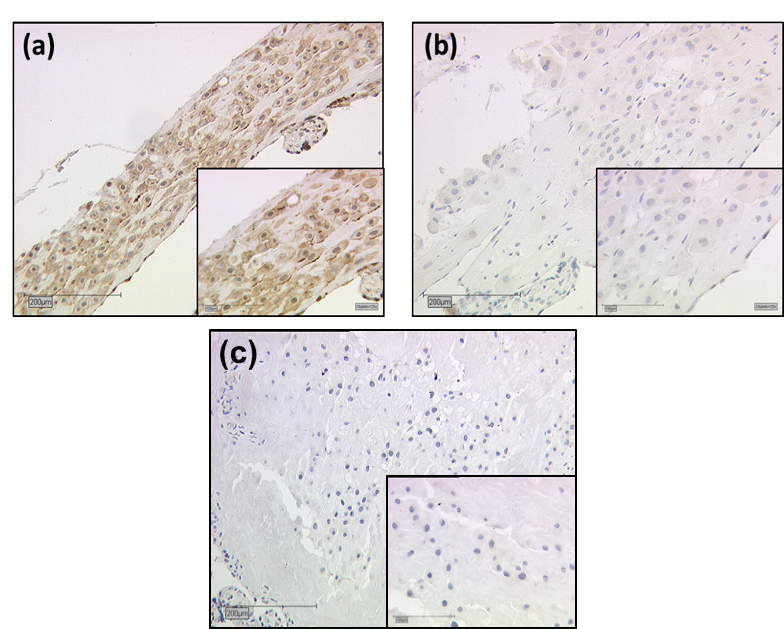


**Supplementary Figure 1:** Positive control staining of EP2 with a magnification of 10× and an insert of 25× EP2, high stained placenta tissue, extra villous trophoblast area (a); EP2 negative control, unstained placenta tissue, extra villous trophoblast area (b); IgG negative control, unstained placenta tissue, extra villous trophoblast area (c).
